# Acute intestinal obstruction due to extrinsic compression by previa myoma and ectopic pregnancy: a case report

**DOI:** 10.1186/s13256-017-1540-8

**Published:** 2018-01-16

**Authors:** Harissou Adamou, Ibrahim Amadou Magagi, Souleymane Oumarou Garba, Oumarou Habou

**Affiliations:** 1 Department of General Surgery, Faculty of Health Sciences, University of Zinder, National Hospital, PB: 656, Zinder, Niger; 2 Department of General Surgery, Zinder National Hospital, PB: 155, Zinder, Niger; 3 Mother-child Center of Zinder, PB: 155, Zinder, Niger; 4 Department of Pediatric Surgery, Faculty of Health Sciences, University of Zinder, National Hospital, PB: 656, Zinder, Niger

**Keywords:** Acute bowel obstruction, Ectopic pregnancy, Compressive myoma, Hysterectomy

## Abstract

**Background:**

Acute intestinal obstruction during pregnancy is a rare digestive surgical emergency with significant maternal and fetal mortality. Diagnosis is difficult, often delaying the management. Here, we report an exceptional association of mechanical acute intestinal obstruction due to compression by previa uterine leiomyoma, and a ruptured ectopic pregnancy.

**Case presentation:**

This is the case report of a 43-year-old primiparous black woman from a rural area, who was admitted to the surgical emergency department for acute intestinal obstruction.

At examination on admittance, our patient had a bad general condition with clinical anemia. She had an occlusive syndrome that had been evolving for 3 days. A physical examination of her abdomen showed a widespread distension with an irregular and polylobed solid mass occupying the whole of the lower-umbilical and hypogastric area. A rectal examination found an empty rectum, and the mass was perceptible in Douglas’s pouch. At the vaginal examination, we found the same mass and a finger holster was clean. The diagnosis of intestinal occlusion by a tumor was retained. The laparotomy revealed a distended intestine, a ruptured right tubal ectopic pregnancy and a polymyomatous uterus. The most massive previa leiomyoma was adhering and compressing the rectal and sigmoidal hinge. A total hysterectomy was performed and histopathological examination of specimens confirmed myoma and ectopic pregnancy. The surgical follow-up was uneventful, and our patient was discharged on postoperative day 12.

**Conclusions:**

The etiological diagnosis of acute intestinal obstruction during pregnancy is not easy, especially in the context of a low-income country where the means of biological and radiological diagnosis are lacking. A laparotomy is required before diagnosis of acute surgical abdomen and its management will depend on the intraoperative findings and the condition of the patient.

## Background

Gastrointestinal surgical emergencies in pregnant women are relatively unusual and present diagnostic problems [1–3]. Acute intestinal obstruction (AIO) is even less common during pregnancy [4–6]. It may be due exceptionally to large myofribroma [7]. In his practice, the general surgeon may be called upon to manage gynecological emergencies which appear as an acute abdomen or are fortuitously discovered at laparotomy [1–6, 8]. The clinical presentation is not unequivocal. It is even sometimes misleading because of the physiological changes in the pregnancy, leading to diagnostic hesitancy and therefore a therapeutic delay [1, 2, 4]. We report our experience on the management of a patient with ectopic pregnancy and a compressive polymyomatous uterus, discovered during a laparotomy for acute intestinal occlusion.

## Case presentation

We report the case of a 43-year-old black woman admitted to the surgical emergency department for abdominal pain with inability to pass gas or stool, evolving for 3 days. She came from a rural community, without a health care structure, located about 100 km from the urban center. The anamnesis found menarche at 16 years old, an irregular menstrual cycle, a previous gestation and parity about 18 years ago, and a child who died at the age of 1 year. Our patient, divorced for 15 years, had reported an abdominal mass evolving for several years (about 10 years) with chronic constipation. The date of the last menstruation was not known. Our patient concealed any notion of sexual intercourse. On admittance to the surgical emergency department, our patient had a bad general condition and clinical anemia. A physical examination of her abdomen noted a widespread distension with an irregular, polylobed mass occupying the entire umbilical region. The supraumbilical stage was tympanic to percussion with elastic resistance to palpation. The rectal examination found an empty rectum, and the mass was perceptible in Douglas’s pouch. At the vaginal pelvic examination, we found the same mass and a finger holster was clean.

**Fig. 1:**
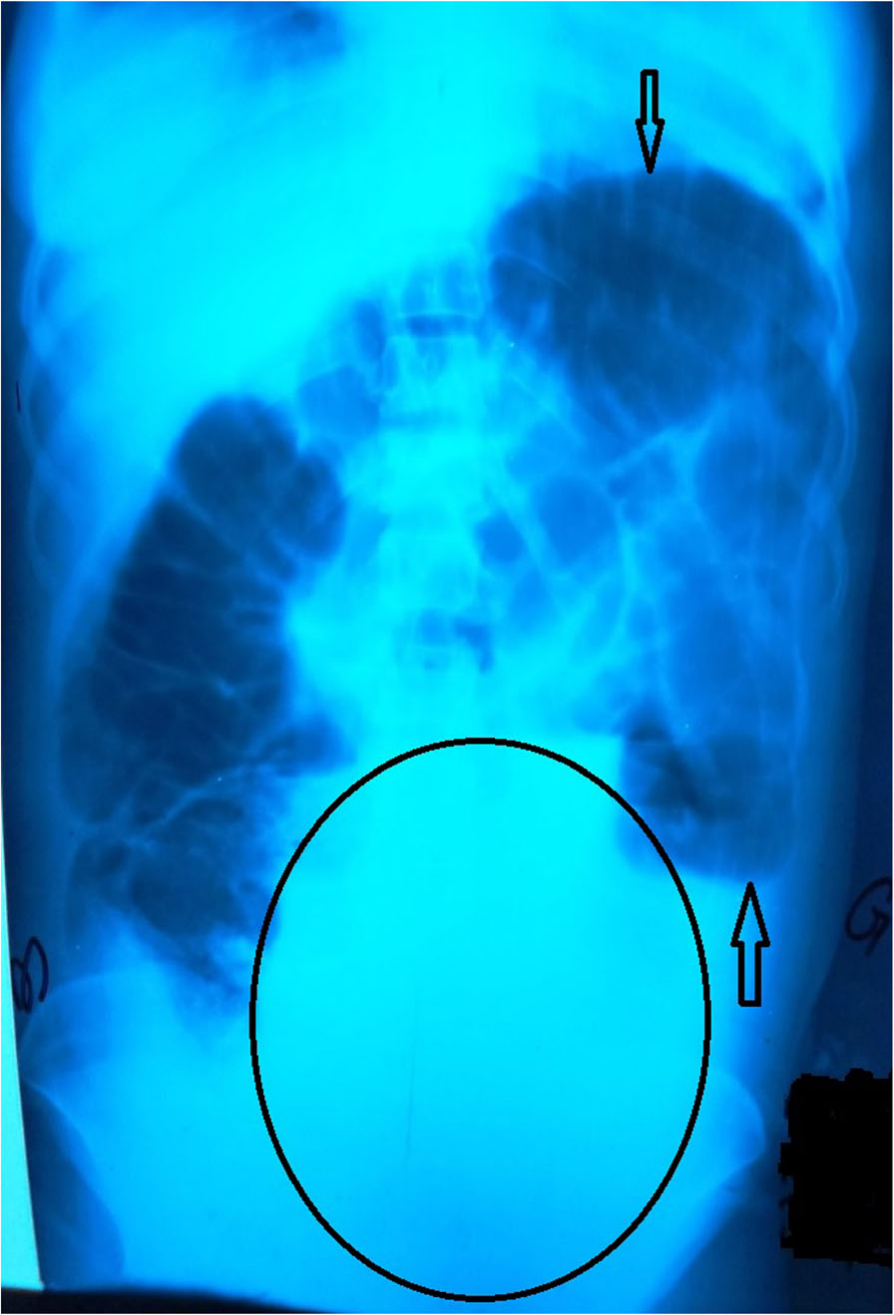
Abdominal X-ray showing intestinal distension with some hydroaerial levels (*black arrows*) and pelvic opacity (*circle*)

An erect abdominal X-ray noted an ileocolic distension with some hydroaerial levels and a pelvic opacity (Fig. 1). The diagnosis of AIO by a tumor was evoked, and emergency laparotomy was indicated. The biological examination noted: anemia at 10 g/dL, and slightly altered renal function (a uremia level of 12 mmol/L, a serum creatinine level of 190 μmol/L).

**Fig. 2:**
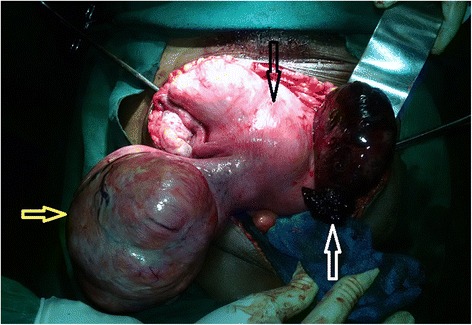
Intraoperative images showing the polymyomatous uterus (*black arrow*), left posterior myoma (*yellow arrow*), and ectopic pregnancy (*white arrow*)

**Fig. 3:**
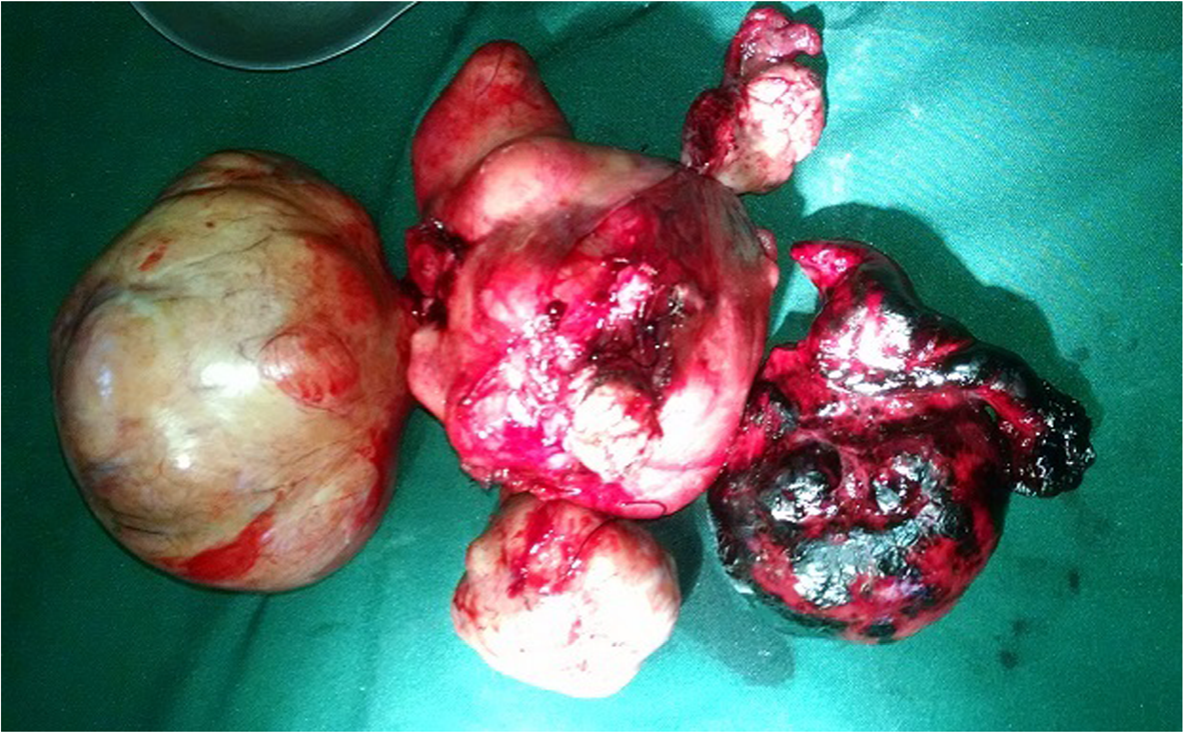
Hysterectomy specimens carrying myomas and appendages (weight = 4.5 kg)

A nasogastric tube, a urinary catheter, and a large venous line were installed for resuscitation. A median laparotomy allowed the aspiration of 1.2 L of blood. Exploration noted a ruptured right tubal ectopic pregnancy and a polymyomatous uterus. The largest myoma previa adhered to the rectosigmoid hinge and compressed it (Fig. 2), explaining the extrinsic obstruction of the colon. A total hysterectomy was performed. The surgical specimen containing the uterus, myomas and annex weighed 4.5 kg (Fig. 3). The most voluminous myoma was 18 cm wide and 23 cm long. The surgical recovery was uneventful, and our patient was discharged on postoperative day 12. Our patient was informed that she could no longer have children. Our patient was very satisfied with the disappearance of this abdominal mass, which hampered her daily activities. A histologic examination confirmed a ruptured ectopic pregnancy and myofibroma without signs of malignancy.

## Discussion

Our patient comes from a rural area. Her admission in a context of surgical digestive emergency led to minimum examinations and the realization of an emergency laparotomy. The limits of this management remain the inadequacy of the diagnostic capability and the difficulty of accessibility to the sanitary structures, resulting in late consultation. But the advantage of the emergency intervention was the discovery and treatment of the ruptured ectopic pregnancy and massive myomas.

Abdominal-pelvic pain, a frequent reason for emergency consultation in pregnant women, may have several origins, obstetric, urological, or digestive [1, 2, 7, 9, 10]. AIO during pregnancy is a rare digestive surgical emergency [4–6, 9, 10]. Its incidence varies between 1 out of 3600 and 1 out of 66,431 pregnancies [1, 5, 6, 9]. The misdiagnosis of AIO during pregnancy leads to high maternal-fetal morbidity and mortality [5, 6]. In most cases, the AIO is linked to flanges or adhesions (in the case of a history of abdominal surgery), intestinal volvulus, intussusception, strangulated hernias, or inflammatory or neoplastic processes [2–6, 9, 10]. The diagnostic difficulty of AIO on pregnancy has been reported by several authors [1, 3, 5, 8, 9]. In our case, ectopic pregnancy was unknown and the discovery was intraoperative. AIO is even rarer in the first trimester of pregnancy, as the risk increases with increasing uterine size [1, 9]. During pregnancy, the AIO would be enhanced by the reduction of intestinal peristalsis, the constipation increased by hormonal impregnation, which leads to the hypotonia of the intestinal musculature, and to the anatomical modification of a sequel flange [4, 5]. In our patient, extrinsic mechanical compression by a previa, myoma was responsible for intestinal obstruction. This kind of intestinal obstruction by uterine myoma previa is little described in the literature [7]. This condition occurs predominantly in three periods of pregnancy: from 16 to 20 weeks when the uterus becomes abdominal and pelvic area; from 32 to 36 weeks when the head of the fetus descends into the pelvis, and during the immediate postpartum period by a sharp decrease in the volume of the uterus [1, 4, 5, 9].

The association of ruptured tubal ectopic pregnancy and AIO has also been described by Singh *et al*. [10]. For these authors, AIO was caused by adherence to a small loop on a broken tubal pregnancy; they concluded that it was difficult to establish an etiologic diagnosis of AIO during pregnancy [10]. The absence of vaginal bleeding and anamnestic data related to pregnancy led us to AIO on tumor, based on occlusive syndrome, palpation of an abdominal mass and radiological images (X-ray). Peroperative incidental discoveries in emergency digestive surgery are usual when modern imaging (computed tomography scan, magnetic resonance imaging) is not available [8]. In the case of an acute AIO-type surgical abdomen, indications of medical or surgical treatment are not affected by pregnancy [1, 2, 9]. Ruptured ectopic pregnancy alone can be responsible for intestinal obstruction due to ileus associated with hemoperitoneum or intestinal adhesions [10]. In the case of our patient, the mechanical compression by the myoma of the rectal and sigmoidal hinge was evident intraoperatively. In the sub-Saharan context, with limited resources, the initial diagnosis of ectopic pregnancy can be very difficult at the initial admission of the patient, especially if the classic signs are not found (irregular menstrual cycles, absence of metrorrhagia, concealment of possible sexual intercourse outside of a conjugal home in a patient divorced for 15 years). Some authors recommend education of other specialists in the detection of ectopic pregnancy in all women of childbearing age in order to reduce its mortality [10, 11].

The management of this emergency is often associated with a delay in diagnosis and is therefore therapeutic, aggravating the maternal and fetal prognosis [1, 5, 9].

In our context, taking into account the age of the patient (premenopausal), the difficulty of access to health facilities, the subsequent risk of developing another ectopic pregnancy on the left fallopian tube, operational difficulties, hemorrhagic risk on the polymyomatous uterus, and risk of malignancy, we opted for a total hysterectomy. In perimenopause and apart from any desire for pregnancy, hysterectomy is the most effective way to manage a voluminous and symptomatic myoma to prevent short or even other long-term complications [8, 12]. It is the alternative that provides an improvement in terms of better comfort of life and sexuality [12]. Some authors [7, 10] opted for conservative treatment for younger patients. Brazet *et al*. [7] performed a delayed myomectomy 6 weeks after cesarean section delivery in a 33-year-old woman at the first pregnancy, thus adhering to the recommendations of learned societies [12, 13].

Intestinal obstruction has various etiologies. In a woman, gynecological causes that present as digestive surgical emergency must be kept in mind.

## Conclusions

The etiologic diagnosis of AIO during pregnancy is difficult. In the Nigerien context, characterized by the unavailability of modern imaging equipment, hesitation has no place in the face of an obvious diagnosis of an acute surgical abdomen of the woman. Thus, laparotomy is necessary, and the surgical procedure must take into account the peroperative findings, the age, the antecedents, and the patient’s desire of pregnancy.
